# A projection of primary knee replacement in Denmark from 2020 to 2050

**DOI:** 10.1080/17453674.2021.1894787

**Published:** 2021-03-08

**Authors:** Louise Daugberg, Thomas Jakobsen, Poul Torben Nielsen, Mathias Rasmussen, Anders El-Galaly

**Affiliations:** a Department of Clinical Medicine, Aalborg University ;; b Interdisciplinary Orthopaedics, Aalborg University Hospital , Denmark

## Abstract

Background and purpose — The incidence of knee replacements (KRs) has increased in the past decades. Previous studies have forecast a continuous and almost exponential rise in the use of KRs, but this rise must cease at some point. We estimated when and at what incidence the use of KRs will plateau in Denmark.

Patients and methods — We retrieved 138,223 primary KRs conducted from 1997 to 2019 from the Danish Knee Arthroplasty Registry. Censuses from 1997 to 2019 as well as population projections from 2020 through 2050 were collected from Statistics Denmark. We applied logistic and Gompertz regression analysis to the data to estimate the future incidence until 2050 with root mean squared error (RMSE) as a quantitative measurement of the models’ fit.

Results — The Danish incidence of KRs from 1997 to 2009 increased by more than 300%, but has stalled since 2009. Logistic and Gompertz regression had an RMSE of 14 and 15 indicating that these models fitted the data well. Logistic and Gompertz regressions estimated that the maximum incidence will be reached in 2030 at 250 (95% prediction interval [PI]) 159–316) KRs per 10^5^ or in 2035 at 260 (PI 182–336) KRs per 10^5^, respectively.

Interpretation — The Danish incidence of KRs seems set to plateau within the coming decades. Countries experiencing a current exponential rise at a lower incidence may benefit from this study’s projection when forecasting their future demand for KRs.

Only few studies have attempted to project the future demand for knee replacements (KRs) (Kurtz et al. 2007, Culliford et al. 2015, Patel et al. 2015, Guerrero-Ludueña et al. 2016) with only 1 conducted in a recent Swedish population (Nemes et al. 2015). Most of these studies are based on historical data from a period when the countries experienced a rapid, almost exponential, growth in the incidence of KRs. Historically Denmark has experienced a similar growth, but within the last decade the increase in incidence has stalled. In all countries, a similar stagnation is to be expected. Yet, when a country is experiencing a rapid increase in the incidence of KRs it is difficult to reliably estimate at which timepoint and volume the incidence will stagnate. Therefore, we used the stagnating incidence in Denmark to make a more reliable estimation of when and at what volume the incidence of primary KRs will plateau during 2020 to 2050.  

## Patients and methods

### Study design

This is a cross-sectional study based on data from primary KRs conducted in Denmark from 1997 through 2019 and presented in accordance with the STROBE Statement.

### Data source

The study was based on the Danish Knee Arthroplasty Registry (DKR), which has monitored KRs conducted in Denmark since 1997 (Pedersen et al. 2012). Within the first decade (i.e., 1997–2007) the completeness of primary KRs in the DKR rose steadily and since 2007 the completeness has been above 90%. The DKR is linked to the Danish National Patient Registry (DNPR), which is the major administrative database in Denmark collecting information on all medical treatments conducted at public and private hospitals since 1968 (Schmidt et al. 2015). This linkage was used to adjust for unregistered primary KRs in the DKR, and thus the total number of primary KRs was in accordance with the DNPR, whereas the reported subtypes of KRs (e.g., total knee arthroplasties or unicompartmental knee arthroplasties) were based solely on the registered KRs in the DKR.

Censuses from 1997 to 2019 and population projections from 2020 through 2050 were collected from Statistics Denmark. Statistics Denmark is the central Danish authority of collecting, processing, and publishing statistical information e.g., censuses in Denmark (Statistics Denmark; https://www.dst.dk/en). The data used in this study was collected on March 9, 2020.

### Study cohort

**Figure 1.**
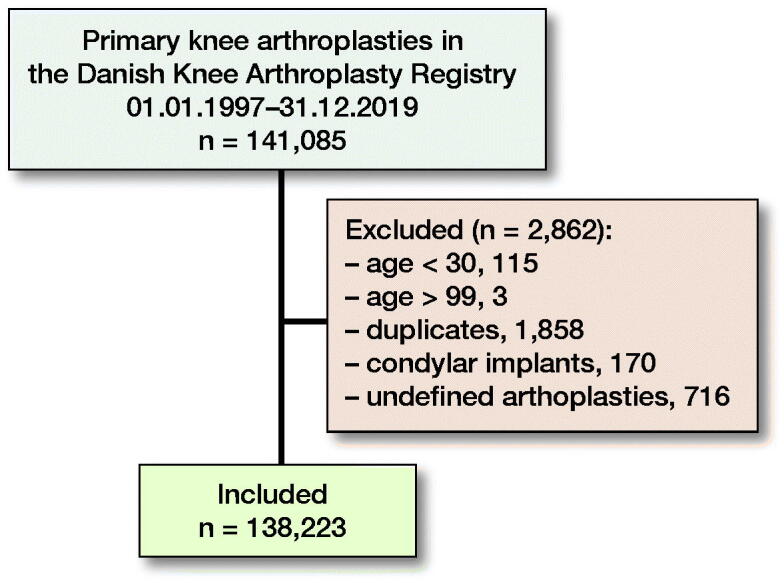
Inclusion and exclusions of study cohort.

From the DKR, we retrieved information on all primary KRs conducted from 1997 to 2019. Individuals younger than 30 or older than 99 were excluded as they do not represent the typical patient undergoing KR. After exclusion of these as well as duplicates, undefined implants, and condylar implants (e.g., hemicaps), 138,223 primary KRs were included in the study cohort (Figure 1).

### Data analyses and modelling

The KRs were divided by type of arthroplasty to see the evolution in the use of different KRs from 1997 to 2019.

The censuses from Statistics Denmark were used to determine the incidence per 10^5^ of primary KRs from 1997 through 2019.

From the incidence and population projections, we estimated the annual number of primary KRs to be conducted in Denmark from 2020 through 2050.

The annual incidence of KRs was regressed on each calendar year from 1997 throughout 2050 with the use of logistic and Gompertz regression analysis on the actual incidences between 1997 and 2019. Logistic regression assumes that the quantity of knee replacements increases in a similar fashion to an exponential curve but gradually slows to linear growth. Gompertz regression analysis assumes that the quantity of knee replacements increases similarly to the logistic model, but the upper asymptote is approached more gradually than in the logistic, where the curve is symmetric.

### Statistics

Root mean squared error (RMSE) was used as a quality estimator of the models’ fit to the data points and used to pick the best-fitted models. All estimates were rounded to the nearest hundreds or thousands (when the projected numbers exceeded 1.000), and presented with their 95% prediction interval (PI).

All analyses were conducted in JMP Pro 15 by SAS (SAS Institute, Cary, NC, USA).

### Ethics, funding, data sharing, and potential conflicts of interests

This study was approved by the North Denmark Region (ID: 2019-107) and by the Steering Committee of the Danish Knee Arthroplasty Registry (DKR-2019-10-16). The study was financed by Interdisciplinary Orthopedics at Aalborg University Hospital. All the data used in this study can be retrieved from the Danish Knee Arthroplasty Registry and Statistics Denmark. All data retrieved from these sources and used in this study can be seen in Table 1. None of the authors report any conflicts of interest. 

**Table 1.** Baseline data and characteristics

Year	Population age 30–99	Primary KR in the DKR	Partial KR	Complete- ness (%)	Absolute number of primary KR	Incidence per 10^5^ Danes
1997	3,274,026	1,386	29	69	2,003	61
1998	3,302,439	1,869	40	83	2,242	68
1999	3,325,149	1,816	46	77	2,373	71
2000	3,343,867	2,235	92	89	2,522	75
2001	3,363,911	2,690	98	87	3,105	92
2002	3,386,844	3,599	220	83	4,339	128
2003	3,409,437	3,940	333	87	4,549	133
2004	3,428,946	4,209	381	84	5,009	146
2005	3,449,794	4,686	414	83	5,616	163
2006	3,471,898	5,445	479	88	6,204	179
2007	3,489,318	7,003	594	93	7,504	215
2008	3,507,360	6,987	707	91	7,651	218
2009	3,528,810	8,219	788	97	8,430	239
2010	3,544,098	8,309	901	96	8,670	245
2011	3,559,168	8,030	888	97	8,278	233
2012	3,570,344	8,042	784	97	8,299	232
2013	3,583,952	7,994	806	97	8,209	229
2014	3,598,842	8,240	897	98	8,394	233
2015	3,619,744	8,180	1,214	98	8,307	229
2016	3,646,223	8,067	1,462	98	8,264	227
2017	3,671,269	8,159	1,727	97	8,384	228
2018	3,695,198	9,240	1,823	97	9,535	258
2019	3,719,214	9,878	2,263	97 **^a^**	10,184	274

KR, knee replacement

DKR, the Danish Knee Arthroplasty Registry

**^a^** Assumed completeness due to a lack of data.

## Results

The annual number of primary KRs conducted in Denmark has increased exponentially, from 2,003 in 1997 to 7,651 in 2008. Since 2008, the increase has gradually stalled and in 2019, 10,184 primary knee replacements were inserted in Denmark (Table 1). The mean patient age at knee replacement surgery in 2019 was 68 years (30–99) and 57% were females. The proportion of unicompartmental knee arthroplasties (UKAs) has increased within the past 2 decades and currently constitutes 22% of primary knee replacements in Denmark (Table 1).

**Figure 2.**
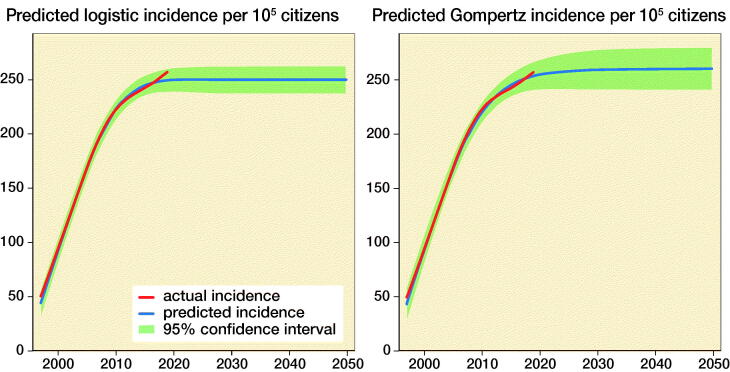
Logistic and Gompertz regression analysis of primary KR incidence per 10^5^ citizens based on data from the DKR between 1997 and 2019. The red line is the actual incidence of primary KRs per 10^5^ individuals from 1997 to 2019, while the blue line is the regression from 1997 to 2050. The green areas signify the 95% confidence intervals.

The logistic and Gompertz regression analysis (Figure 2) had an RMSE of 13.9 and 15.5 respectively, which represent an acceptable fit and thus these models were used for forecasting. The regressions forecast a maximal incidence at 250 (PI 159–316) per 10^5^ in 2030 (logistic regression) or 260 (PI 182–336) per 10^5^ in 2035 (Gompertz regression) (Table 2). However, as the population grows the annual number of primary KRs continues to increase until 2050 (Table 2) when between 10,379 (logistic regression) and 10,808 (Gompertz regression) are expected.  

**Table 2.** Projection results based on logistic and Gompertz regression of the primary KR incidence per 10^5^ Danes between 1997 and 2050

		Logistic regression		Gompertz regression	
	Population age 30–99	Incidence (PI)	Predicted number of primary KRs	Incidence (PI)	Predicted number of primary KRs
2000	3,343,867	86 (79–239)	2,878	90 (80–236)	3,009
2005	3,449,794	172 (93–252)	5,947	172 (95–250)	5,937
2010	3,544,098	226 (106–264)	8,004	221 (109–264)	7,850
2015	3,619,744	244 (120–277)	8,819	244 (124–278)	8,844
2020	3,745,142	248 (133–290)	9,298	254 (139–293)	9,506
2025	3,881,337	249 (146–303)	9,679	258 (153–307)	9,999
2030	3,990,028	250 (159–316)	9,961	259 (168–322)	10,339
2035	4,061,503	250 (172–329)	10,142	260 (182–336)	10,548
2040	4,116,730	250 (184–343)	10,280	260 (196–351)	10,700
2045	4,135,043	250 (197–356)	10,326	260 (210–366)	10,752
2050	4,156,132	250 (209–370)	10,379	260 (223–381)	10,808

The estimations of annual numbers were based on the incidence regressions along with the predicted population size in the given year.

## Discussion

We found that the incidence of primary knee replacement will plateau within the next 10–15 years with an expected maximal annual incidence between 250 and 260 per 10^5^ Danes.

Comparable studies from the USA, United Kingdom, Spain, Australia, Germany, and Sweden have either found no maximal incidence within their projection, a higher maximal incidence per 10^5^ citizens than presented in our study, a slowing incidence rate (Bini et al. 2011), or the nearing of what seems to be a maximal incidence. The higher maximal incidences in the aforementioned studies might be due to wider inclusion of KRs, such as revisions, or difficulties estimating the maximal incidence at the time of an exponential or linear rising incidence (Kurtz et al. 2007, Culliford et al. 2015, Nemes et al. 2015, Patel et al. 2015, Guerrero-Ludueña et al. 2016, Inacio et al. 2017a, 2017b, Rupp et al. 2020). It must be noted that a plateau in the annual number of primary KRs seems to be ensuing in the study by Guerrero-Ludueña et al. (2016); however, data from the private sector was not included, therefore the forming of a plateau can neither be confirmed nor denied.

Niemeläinen et al. (2017) investigated the different incidences of knee arthroplasty in Denmark, Sweden, Norway, and Finland from 1997 through 2012. They found that the incidences of primary TKA and UKA per 10,000 inhabitants over the age of 30 increased in all 4 countries: Denmark (3.4–21), Sweden (9.0–21), Norway (3.6–14), and Finland (13–28). However, they assumed a 10–15% underestimation of the Danish data due to lower completion of the DKR in the first 10 years of the study period. At the end of their study period, Danish and Finnish incidences seemed more stable in nature than the Swedish and Norwegian incidences, which still showed signs of linear to exponential growth. This is the start of the bend in the Danish incidence curve observed from 2010 through 2017. This had not been observed in other countries at the time of forecasting, which might be the reason why this study found that the projected incidence of primary KR in Denmark will reach a plateau sooner than the previously reported forecasts from comparable countries. Naturally, this bend in the Danish incidence curve from 2010 through 2017 could constitute a halt in growth, which would be replaced by a continuous future rise. However, the duration of the stable incidence counters that it should be outlier-years.

The soon-to-be reached Danish maximal incidence might in part be due to the Danish tax-paid healthcare system, in which patients’ personal economy is not a limiting factor for healthcare accessibility. In practice, this means that every person assessed to require a KR will be offered one. Additionally, the required sick leave for the surgery and rehabilitation are paid for by the Danish Healthcare System, as are many other additional needs. The high accessibility and equality in Danish healthcare also ensures the high external validity of our study (Schmidt et al. 2019). The annual number of Danes needing a KR is comparable to the annual number of Danes receiving a KR. This results in higher incidences (the Danish KR incidence was the 10th highest among the OECD countries in 2017 (Organization for Economic Co-operation and Development 2019) compared with countries thay have more privatized healthcare or less equal healthcare, since some individuals will not be able to afford the surgery or unpaid sick leave. This leaves a group of unknown size without representation in future projections. Another reason for higher incidences in Denmark might also be a result of patient preferences, since hindrances to surgery are few in Denmark. For example, the waiting time for KR in Denmark was the lowest among the OECD countries in 2017 (Organization for Economic Co-operation and Development 2019). This in essence means that the incidence of KR in Denmark might be closer to the actual need for KRs compared with countries with privatized healthcare.

Our study has some limitations. 1st, projections are sensitive to changes in the population, treatment protocols, or trends in treatment. Trends such as surgeons relying on partial knee replacements, e.g., medial unicompartmental arthroplasty to a higher degree than earlier (see Table 1) or changes in treatment protocol towards more or less conservative treatment, are also liable to affect future incidences.

2nd, several implants were excluded due to duplication, a lack of description, etc. before an adjustment with data from the NDPR was made, in order to create realistic estimates of the annual burden of KR. This might have resulted in less precise regressions; however, the difference would be minimal. 3rd, we were unable to retrieve projections of the evolution of known risk factors for OA such as BMI. Countries with either a higher mean BMI or a more rapid increase in their mean BMI might experience a more rapid increase in the need for KRs or higher maximum incidence than we found.

In conclusion, the incidence in Denmark of primary knee replacements seems to be nearing its plateau, but in spite of this the absolute number of primary KRs will continue to increase as the population gets older. The Danish healthcare system ought to prepare for an increase in primary knee replacements as well as revisions in the future.
